# Round the Hospitals

**Published:** 1916-12-09

**Authors:** 


### ROUND THE HOSPITALS.

It is of immense importance to the whole body of British nurses that the authorities of the great hospitals and nurse-training schools in Scotland, who are members of the Association for the Promotion of the Registration of Nurses in that country should have united in favour of the College of Nursing and the Hon. Arthur Stanley's Bill. The names attached to the protest of this Association, which we published last week, page 188, demonstrate its overwhelming strength across the Border. They further exhibit the fulness of knowledge and far-reaching experience which have called forth the Association's protest against the unconstitutional proceedings of the Central Committee for the State Registration of Nurses in London. In view of the Scotch protest, these proceedings must be reconsidered by a special meeting of the whole of the members of the Associations represented on this Central Committee, or the Central Committee for the State Registration of Nurses in London must necessarily cease to carry further weight in the nursing world or in Parliament.

Christmas in the voluntary hospital has long been for the majority of nurses a day of rejoicing and blessedness. The initial preparations, the continuous hard work consequent upon full beds, wherein old patients not infrequently contrive to find a place, the decorations, the entertainments, the raising of means to make each ward at its best, all combine to put extra pressure on the sisters and nurses. We have often said that a voluntary hospital is the best place for a man or woman of heart to visit on Christmas Day. There is fellowship, inspiration, the evidence that everybody is unselfishly devoting him or her self to the personal service of bringing brightness and hope and forgetfulness and joy into the hearts of the sick and the patients generally, not forgetting the children. All this cannot fail to touch the heart of the dullest of visitors, and possibly even the hidebound routine of introspective selfishness.

---

Amid all this scene of brightness and life the moving spirits round the matron and officers are the sister and the nurses, upon whom the lion's share of duty and work devolves. The *Nursing Mirror* this week publishes an application form for nurse registration, and invites each of its nurse readers to make a point to fill up the form, sign it, and send it to the Editor on or before Christmas Eve as a present for herself and a handshake for the Editor. In view of the policy of disunion adopted by the

Central Committee for Registration, it is essential that nurses should range themselves for defence, and without delay stand up for their rights, by uniting in upholding the College under Mr. Stanley, their Parliamentary leader. There should be no difficulty, if this is done, for 20,000 nurses to register with the College of Nursing before February is out. Then the College of Nursing and the system of nurse registration can be made accomplished facts within 1917. Every sister and every nurse owes it to herself to find the time to fill in an application form for registration, and to post it to the Editor of the *Nursing Mirror* by Christmas Eve at the latest. Those wise virgins in the nursing world who do this can be freed from all worry as to the payment of the registration fee, for such payments will be adjusted through the Editor to suit the convenience of each nurse. We hope for the co-operation of the matrons and superintendents throughout the country with the *Nursing Mirror* in making Christmas 1916 memorable in the history of the College of Nursing.

